# Transient Hydroureteronephrosis Caused by a Foley's Catheter Tip in the Right Ureter

**DOI:** 10.1100/tsw.2005.45

**Published:** 2005-05-02

**Authors:** Jacob George, George Tharion

**Affiliations:** Department of Physical Medicine and Rehabilitation, Christian Medical College, Vellore, Tamil Nadu, India

**Keywords:** spinal cord injury, urethral catheterization, hydroureteronephrosis

## Abstract

We report a case of unilateral hydronephrosis following urethral catheterization in a patient with T6 complete paraplegia at the Physical Medicine and Rehabilitation Department in a tertiary care teaching hospital, India. Diagnosis was established by an abdominal ultrasound. The misplaced catheter tip was withdrawn from the ureteric orifice and hydronephrosis was resolved. Foley's catheterization, a widely practiced clinical procedure, is not without its attendant risks of an inadvertent placement in the ureter leading to transient hydronephrosis. Inadequate drainage through a catheter should thus alert one to this potentially hazardous complication that can be diagnosed by an early ultrasound. This complication can be avoided by gently tugging on the catheter after inflating the catheter bulb.

## INTRODUCTION

Several methods are described in the literature for the management of acute and chronic neuropathic bladders[1,2,3,4]. Clean intermittent catheterization (CIC) is the most acceptable mode of early urologic management of a patient with acute spinal cord injury[5], however, it is a common clinical practice to catheterize the bladder following acute spinal cord injury and is occasionally performed as a medical emergency for urinary retention. The difficulty in training the patient or the caregiver in the emergency room to perform CIC is one of the ascribed reasons for this practice. The common complications observed following urethral catheterization are urethral trauma, inflation of catheter bulb in urethra, entry into false passages, and catheter blockade. Rare complications reported in the literature include intravesical knot of the Foley catheter and rupture of the bladder or urethra[6,7,8,9]. Acute hydronephrosis following neuropathic bladder is managed by providing continuous bladder drainage using an indwelling urethral catheter[10]. We report a rare case of hydroureteronephrosis produced by the placement of the Foley catheter tip into the right ureter in a paraplegic patient during urethral catheterization.

## CASE REPORT

A 14-year-old boy presented to us with T6 paraplegia following Acute Demyelinating Encephalomyelitis (ADEM). His bladder was being drained by an indwelling urethral catheter. During the course of treatment, this catheter was changed and replaced with a new 16 Ch Foley catheter. Within 24 h of the catheter change, he presented with right-sided abdominal discomfort and leakage of urine along the side of the urethral catheter. An ultrasound examination of the abdomen was done, which revealed the tip of the catheter located inside the right ureter with edema of the vesicoureteric junction (Fig. A) and unilateral hydroureteronephrosis (Fig. B) on the right side. The catheter was immediately repositioned and the patient's symptoms subsided. A repeat ultrasound examination showed normal kidneys and ureters with a bifid renal pelvis on the right side (Fig. C). A cystoscopic examination performed 2 weeks later showed a normal vesicoureteric junction. A cystometrogram was also done, which showed small-capacity bladder with detrusor hyperreflexia.

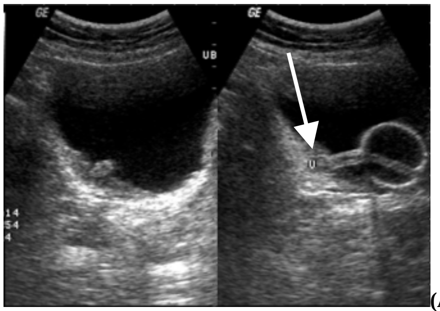

(A)

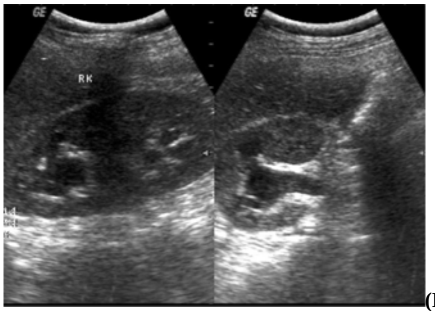

(B)

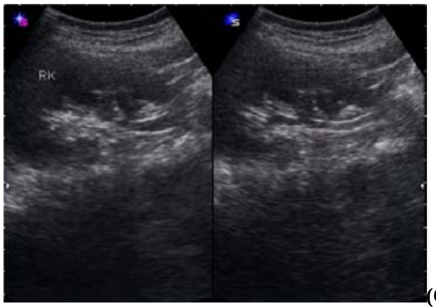

(C)

**FIGURE**. Abdomen ultrasound showing (A) tip of the Foley's catheter inside the right ureter (arrow), (B) right hydroureteronephrosis, (C) hydroureteronephrosis subsided after repositioning the catheter.

## REVIEW OF THE LITERATURE

Kato[11] has reported the inadvertent placement of a catheter in the left ureter in a small contracted urinary bladder with patulous ureteric orifice. Cuttino and Clark[12] also reported bilateral ureteral obstruction caused by a Foley balloon in a patient with a contracted bladder. Gill and Nichols[13] reported a similar accidental placement of a catheter during cystometry. In their report, the vesical pressure rose to 150-cm water and the patient developed colicky abdominal pain and on repositioning the catheter, the cystometry showed a normal vesical pressure. Borrero et al.[14] reported a case of acute obstruction at the ureterovesical junction caused by a suprapubic cystostomy catheter tip. Singh and Eardley[15] reported a case of Foley's catheter entering the retroperitoneum, along a longitudinal tear in the right ureter, caused by upward migration of the urethral catheter.

## DISCUSSION

The inadvertent placement of a urethral catheter into the ureter through a patulous vesicoureteric orifice has been reported in the literature. In our patient, the small-capacity bladder could have contributed to the placement of the catheter tip in the ureter. As the draining tip of the catheter was in the right ureter, the urine from the left ureter had to find its way out along the side of the indwelling catheter. The right ureteric orifice was occluded by the catheter tip, resulting in hydroureteronephrosis on that side. As the paraplegia was at upper thoracic level, the patient was vulnerable to complications from autonomic dysreflexia triggered by urinary tract obstruction. However, he did not show any evidence of this complication.

Foley's catheterization, a widely done clinical procedure, is not without its attendant risks. Inadequate drainage through the catheter could be due to the catheter tip not being in the bladder and an early ultrasound could reveal this potentially hazardous complication. This inadvertent placement of the urethral catheter could possibly be avoided by gently tugging on the catheter after catheter bulb inflation following catheterization. Inadequate urinary drainage following urethral catheterization should alert the team to look for possible complications including a catheter tip being misplaced into the ureter. Persistence of discomfort should warrant investigations including ultrasound to ensure the proper placement of the catheter.
